# “A bit lost”—Living with attention deficit hyperactivity disorder in the transition between adolescence and adulthood: an exploratory qualitative study

**DOI:** 10.1186/s40359-024-01522-1

**Published:** 2024-01-11

**Authors:** Inger Lise Rasmussen, Jorun Schei, Kristin B. Ørjasæter

**Affiliations:** 1 Nord Trondelag Hospital Trust, Namsos Hospital, Namsos, Norway; 2 NTNU, St. Olavs University Hospital, Trondheim, Norway; 3 Faculty of Nursing and Health Science, Nord University, Namsos, Norway

**Keywords:** ADHD, Adolescents, Emerging adulthood, Transition, Psychosocial interventions, Recovery

## Abstract

**Background:**

Attention deficit hyperactivity disorder (ADHD) begins in childhood and in many cases persists into adulthood. The transition from adolescence to adulthood for young people with ADHD is a vulnerable time and can be associated with comorbid conditions and unfavorable outcomes. Thus, further studies are needed to explore the characteristics of the transition period in emerging adulthood. The overall aim of this study was to gain increased knowledge of emerging adults’ experience of living with ADHD in the transition from adolescence to adulthood. This is a follow-up from a previous qualitative study that examined how young people experience receiving and living with a diagnosis of ADHD.

**Method:**

The study has a qualitative retrospective design. Seven participants were included in this study using a purposive sampling method. We re-invited the same participants who were interviewed in 2015–2016 and conducted in-depth interviews. The data were subjected to Malterud’s systematic text condensation (STC).

**Results:**

Four crosscutting themes were identified from our analysis: (1) low level of knowledge about ADHD and treatment options; (2) barriers to seeking and accessing help; (3) developing self-help strategies; and (4) a preference to discontinued medication use.

**Conclusion:**

The participants emphasized a need for more information about ADHD in transition phases and support, both from professionals and peers, about finding ways to live meaningful lives. The treatment they had been offered was particularly linked to symptom reduction and medication use. A more appropriate focus would have been linked to how they, as citizens, could gain knowledge and skills to live meaningful lives with ADHD.

**Supplementary Information:**

The online version contains supplementary material available at 10.1186/s40359-024-01522-1.

## Background

Attention deficit hyperactivity disorder (ADHD) is a neurodevelopmental disorder that begins in childhood and frequently persists into adulthood. ADHD is clinically defined by core symptoms of inattention, hyperactivity, and impulsivity that cause functional impairments in multiple domains of daily life (ICD-11 and DSM-5-TR). It is not uncommon for people with ADHD to also have co-occurring conditions such as lower self-esteem [1, 2], depression [3], and anxiety disorders [4]. The reported prevalence estimate for ADHD in adults is 2–3% [5].

Emerging adulthood from the age of 18 to 29 is a critical, complex developmental phase, where the gap in the “child–adult system” must be bridged [6, 7], and a critical period of change in most young people’s lives [8]. Among those living with ADHD, this period can be associated with unfavorable outcomes and persisting comorbid problems into adulthood [9–11]. Emerging adults with ADHD may be particularly vulnerable during this transitional phase, as ADHD increases the risk of school dropout and unemployment in adulthood [12, 13]. In addition, emerging adulthood is a peak period for experimentation with substance use [4, 14] and for the onset of mental health problems, including self-harm and suicide [7].

Guidelines worldwide recommend a stepwise approach to ADHD treatment, beginning with nonmedical interventions and adding medical treatment for those most severely affected [15]. However, information on the long-term effect of medical treatment is inconclusive [16, 17], and many young people discontinue medical treatment in the transition to adulthood [16]. Therefore, Titheradge et al. (2022) stated that a multidimensional approach, including psychosocial interventions, is needed to improve long-term prospects for young people with ADHD [18]. Nevertheless, medication and school support were the most usual treatments among children and adolescents in the US, whereas fewer received psychosocial treatment [19].

Psychosocial interventions largely focus on the improvement of ADHD-related impairment, rather than symptom reduction [20]; these interventions are in line with recovery-oriented interventions that aim to enhance well-being and the achievement of personally meaningful goals [21, 22]. Recovery is a central perspective in mental health care, emphasizing a personal, relational, and social process toward living a meaningful life [23, 24]. Nevertheless, the understanding of recovery in Norwegian specialized mental health services is limited [24].

The National Alliance of Mental Illness [25] described psychosocial treatment as various types of psychotherapy as well as social and vocational training intended to provide support, education, and guidance to those living with mental health disabilities and their families. Psychosocial treatment is focused on improving quality of life and limiting difficulties at home, school, and work. Indeed, psychosocial treatment includes numerous interventions, such as behavior management interventions, cognitive behavioral treatments, training interventions, and psychoeducation [26, 27]. Psychological interventions delivered via the internet are becoming more common [28], and, recently, Kenter et al. (2023) found significant improvements in self-reported ADHD symptoms and quality of life related to internet intervention (MyADHD) [29].

Several systematic reviews have been conducted on psychosocial interventions for ADHD in recent years [26, 30, 31]. Despite several reviews, an incomplete picture of the effects of psychosocial intervention on ADHD still remains, and limited studies on adolescents have been done [27]. Some evidence has favored a psychoeducational approach, but little is known about the effectiveness of psychoeducation in young people with ADHD [32]. Psychoeducation is defined as a “systematic and didactic approach, adequate for informing patients about the illness and its treatment” [33]. Powell (2022) found that psychoeducation can improve social skills in young people with ADHD; it is also helpful for adults with ADHD in pharmacological treatment who still have significant symptoms [34].

Due to the increasing range of available options, it is becoming more difficult for clinicians to prioritize treatment strategies for ADHD [35]. However, clinicians should be able to provide timely and appropriate information to emerging adults with ADHD in order to support their transition into adulthood [36]. In addition, young people with ADHD are least likely to make a successful transition to adult mental health services [37], as mental health needs are unmet in young people in general, and it is critical to address mental health so that they can fulfil their potential [37, 38]. The transition period from adolescence to adulthood is a vulnerable one [39]. Therefore, additional studies are needed to explore the characteristics of the transition period [40]; there is potential to improve approaches for the treatment of emerging adults with ADHD [41]. Further, adjustments may improve educational possibilities and adult life outcomes [42].

## Method

### Aim

The overall aim of this study is to increase knowledge of emerging adults’ experience of living with ADHD during the transition from adolescence to adulthood.

Because we were interested in the lived experiences of young people with ADHD in emerging adulthood, the design is an explorative qualitative study. We used Malterud’s systematic text condensation (STC) to analyze the data. STC is inspired by Giorgi’s psychological phenomenological approach and is an analysis strategy that can be used for thematic cross-case analysis of various types of qualitative data, including interview studies [43].

### Participants

This study is a follow-up from a previous qualitative study that examined how young people experience receiving and living with a diagnosis of ADHD [44], where we found that participants` self-esteem was stronger than suggested by previous literature. Further, we became interested in their strengths and aimed in a second article [45] to gain a deeper understanding of their self-esteem. In the present study, we wanted to explore how the same participants coped with life in emerging adulthood.

All participants were previous patients from Child and Adolescent Psychiatric Services in central Norway. We re-invited the same participants who were interviewed in 2015–2016. Eight participants were invited by letter, and we followed up with a phone call because many of them had moved since the first interviews took place. Seven of them volunteered to participate: two females and five males, aged 22–27. Written informed consent was obtained from all participants. They could withdraw from the study without giving a reason. To ensure anonymity, the participants’ names have been altered.

A clinical psychologist or a child psychiatrist established the diagnoses of ADHD, during 2007–2008, when participants of this study were assessed in the outpatient clinic of child and adolescent psychiatry. The clinics followed standardized procedures for assessment and diagnosis based on the Norwegian national guideline for assessment and treatment of ADHD (Norwegian Directorate of Health) [46]. Participants Characteristics, is being presented in Table 1.

**Table 1:** Participants’ Characteristics

Category	Variables	n
**Gender**	Female	2
	Male	5
**Age at interviews**	21-year-old	1
	22-year-old	1
	24-year-old	4
	27-year-old	1
**Diagnosis 2007/2008**	ADHD	7
**Additional diagnosis**	Behavior disorder	1
	Impressive language disorder	1
	Depression	1
**Education/work status**	Working	5
	Working part time	1
	Studying	1

### Data collection

During March–August 2022, we conducted open-ended, in-depth interviews lasting 67 to 95 min, because we wanted to reveal the complexity of the participants’ experiences [47], and we wanted to listen carefully with an open mind so that we could hear new and unexpected stories from the participants [48].

The interview guide was developed for this particular study and has not been published elsewhere. The interview guide consisted of several areas: (1) current experiences centered around living with ADHD from adolescence to adulthood; (2) experiences with education, school, and work; (3) thoughts about self-esteem and meaningful relationships; (4) coping and resilience; and, finally, (4) experiences concerning support and treatment.

Informant selection was based on a purposeful sampling strategy, as appropriate, when identifying participants with particular knowledge, skills, or experiences relevant to the research question [48, 49]. Therefore, to explore the experience of living with ADHD in the transition from adolescence to adulthood, we recruited emerging adults who have lived experience with ADHD in this transition phase. All interviews were audio recorded and transcribed verbatim by the first author. The transcribed interviews contained all words, laughing, crying, and pauses.

### Data analysis

The interview data were analyzed using STC [48]. STC is a descriptive method for thematic cross-case analysis of phenomena, representing an approach inspired by Giorgi’s phenomenological ideas [43]. We strive to obtain the criteria for reporting qualitative research, so we used the COREQ checklist. This is a guide designed to (1) encourage improvement in the quality of reporting of qualitative studies and (2) describe coding and how the researchers perceived, examined, and developed understanding of the data [50].

The analysis consists of the following steps: (1) total impression from chaos to themes; (2) identifying and sorting meaning units from themes to codes; (3) condensation from code to meaning; and (4) synthesizing from condensation to descriptions and concepts [43].

Step 1: Total impression from chaos to themes. Authors 1 and 3 read through all the raw data, took the meta-position and bracketed the preconceptions, kept an open mind about our theoretical framework during the process [48, 51], and took the bird’s-eye view [43]. From the first reading of the entire data, Authors 1 and 3 discussed preliminary themes in a workshop and chose 10 preliminary themes, which were given the following names: coping, not giving up, lack of nonmedical treatment, starting to work, being independent, a platform to shine, “ADHD smart,” finding oneself, stopped taking medication, and stigma. They were discussed in the workshops that Authors 1 and 3 participated in. Then, an analytic reduction took place from 10 to 6 revised themes: (1) lack of alternative/psychosocial treatment; (2) independence, do not ask for help; (3) adversity; (4) stopped taking medication; (5) self-esteem; and (6) how to understand themselves as adults.

Step 2: Identifying and sorting meaning units from themes to codes. We performed a systematic exploration of the material we had identified at this point in the analysis. We identified meaning units, and the first author sorted them into colors. Then, Authors 1 and 3 had workshop meetings to identify codes for further analysis; during this process, we removed the meaning units from their original context through another analytic reduction—more specifically, a systematic decontextualization through systematic abstraction. The codes represented empirical findings and were not identified in advance.

During the analysis process, we discovered that our participants rarely sought help for their challenges, and further examined the data with several questions in mind: What kind of support or help did they describe that they need? What kind of help have they been offered? If they needed help in the transition phase between adolescence and adulthood, why did they not ask for it? What kind of help did they experience as useful? Do they use medication today? These questions were helpful in order to select extracts from the transcripts and to check preliminary findings and interpretations against raw data [52]. These questions helped us discover additional codes and subgroups in the data analysis.

Finally, the main topics from Steps 1 and 2 were reduced to five codes: (1) lack of psychosocial treatment in the transition into adulthood; (2) discontinuation of medical treatment; (3) lack of knowledge about ADHD; (4) need to fix their problems themselves; and 5) self-esteem.

Step 3: Condensation from code to meaning. All of the authors read the meaning units within these five code groups, Authors 1 and 3 discussed them in a workshop, and Author 2 gave digital feedback. Then, we carried out a systematic analytic reduction into four themes. Finally, we sorted the data materials under these themes into subgroups, which became subject to further analysis. Some of the subgroups switched places or were combined during this part of the analysis. Next, we identified the core meaning units and put them into a table. Author 1 wrote a condensate for each subgroup by trying to maintain the participants’ original terminology. Then, we found core quotes for each condensate. We visualized the code groups and subgroups in the analyses within Table 2.

**Table 2:** Code groups and subgroups

Code groups	1. Low level of knowledge about ADHD and treatment options	2. Barriers to seeking and accessing help	3. Developing self-help strategies	4. A preference for discontinued medication use
Subgroups	a. Lack of treatment options b. Need knowledge about how to live with ADHD c. Lack of low-threshold services	a. Need help, but do not seek it out b. Do not ask for help due to stigma	a. Make use of their own resources b. Take care of oneself	a. Wish to discontinue medication b. Discontinued because of side effects

Step 4: Summarizing into themes. We wrote an analytic text, adding the core quotes for each of the code groups with a descriptive headline. These will be our final presented themes, which illustrate the participants’ experiences.

In this study, the authors’ backgrounds may have influenced the analysis, as researchers do not stand outside their research in an objective position [47].

To establish trustworthiness of the findings, the researchers strived to provide tick descriptions through the data analysis [52]. We referred to the process in four steps [48]; however, we moved backward and forward between these steps. In addition, Author 1, read the raw data several times to make sure our findings were derived from the data, and stored all of the raw data on a secure network location. In addition to the workshop debriefings between the researchers, Author 1 also discussed the findings with the user organization (ADHD Norge).

The authors reflect broad clinical and research experience in health sciences. The first author is a trained clinical educational therapist with extended experience in child and adolescent psychiatric services. The second author is a child and adolescent psychiatrist and an associate professor in mental health sciences. The third author is a trained clinical social worker and family therapist in adult mental health services, has research experience in mental health recovery, and holds a PhD in health sciences.

## Results

Four crosscutting themes were identified from our analysis: (1) low level of knowledge about ADHD and treatment options; (2) barriers to seeking and accessing help; (3) developing self-help strategies; and (4) a preference to discontinued medication use.

### Low level of knowledge about ADHD and treatment options

The participants in this study found the transition from adolescence to adulthood demanding. They were unsure about what to expect given that they had an ADHD diagnosis, and they emphasized the need for more knowledge about ADHD and how such a diagnosis can develop in transitional phases of life. Phillip pointed out a particular need for knowledge about ADHD symptoms in the context of why, when, and how:

Just need an explanation [for] why things happen, not just that they do happen: “Yes, you have ADHD, that’s why it happens.” I want to know why! (…) What to predict for a 13-year-old who is starting at secondary school, what to predict at 16 years old, when I start primary school, what to predict at 20 years old, when I start working, what can I expect when I am in my 30s when I am planning a family? What can I expect when I am 50 years old? (Phillip)

Furthermore, the participants wanted access to knowledge and examples on how to handle everyday life and when specific life situations occur when struggling with ADHD. The participants felt this kind of focus was lacking in the mental health services. They sought out detailed guidance about living well with ADHD; however, this need was not available by specialized mental health services. Most participants used social media as an information channel to learn more about ADHD. John chose to follow videos on TikTok from young people describing living with ADHD. He argued that such information is easy to find on social media and that experiences from others with ADHD were useful for him:

TikTok is perhaps exactly what individuals need—not just to be told what to do, but what people actually observe about themselves, like, “Hey, this is a thing that is common when you have ADHD, it works like that, you act like this, and people around you experience it like that.” (…) Just talk about ADHD to people with ADHD…so maybe if you hear about it, you get the idea that “oh yes, of course yes, I never thought about this. I do it all the time; oh yes, so it’s okay, then. I thought I was just being stupid.” (John).

The fact that the participant had received limited knowledge from professionals about living with ADHD prompted them to conduct their own research. As they searched on social media, the quality assurance on the information they acquired was limited.

The participants did not ask for treatment for their ADHD symptoms; rather, they wanted advice on how to live with their symptoms and challenges, to be able to cope with life, regardless of their diagnosis, and to be guided on how to live with ADHD.

### Barriers to seeking and accessing help

Most of the participations had not sought out information or had limited access to treatments suited to their needs of living with and managing their ADHD symptoms. Although they described difficult periods and expressed a need for help and support, they chose to decline follow-up from the specialized mental health services. Oliver was among those who had experienced life challenges and whose private network had suggested professional help, but he decided not to seek out professional help.

I have not been in contact with help services or similar.…However, you know life, there are ups and downs all the time, and people who are close to me have suggested…that I could contact a psychologist, you know, just to talk a little bit. (Oliver)

Tom was the only participant who received psychotherapy for emotional regulation before he turned 18 years old. Nevertheless, he felt “too normal” to receive treatment from specialized mental health services; due to stigma, he did not feel comfortable receiving it within specialized mental health services as he did not associate with other patients who received treatment in this system.

I seriously think that it should be easier to get help. When you go into a psychiatric clinic, well, you are not.…I think it is a bit shameful because when I sat in the waiting zone, and saw all the others who passed by, there was obviously something wrong with them, but I didn’t feel like there was something wrong with me. (Tom)

If the follow-up had been at a lower level of care, it could be easier to accept help, as there is a greater chance that he associate himself with the others who receive help.

All participants had received medical help from specialized mental health services during adolescence. However, in the transition between adolescence and adulthood, some called for support and help with a lower threshold, whereas others denied that they would accept any treatment offer.

Nevertheless, they found it difficult to accept or seek out help from others and wanted to cope by themselves; this was also the case for Phillip. At the age of 25, he had never taken the initiative to seek out help for himself:

There is one thing for sure: I honestly can tell with my hand to my heart, for me, it is impossible; I will never seek help for myself. Even though I understand deep down that I need help, especially when living with an ADHD diagnosis from childhood…I would have never done it; I definitely would not have asked for help. I will go on with my misery, I guess, which is slightly the truth, regarding my mental health. (Phillip)

### Developing self-help strategies

The participants shared stories about how they made use of their own resources and took care of themselves throughout their challenges. However, they revealed that they needed practical support and skills training related to living with ADHD. Phillip had high symptom pressure, but he did not want external help with symptom reduction. Instead, he wanted to figure out on his own how to deal with ADHD symptoms in his everyday life.

I feel that it is all about the self-esteem you gain in the end.…I might have to work twice as hard…but I do not want anyone to hold my hand to the finish line.…I have to do it on my own terms. (Phillip)

Participants wanted to choose their own treatment; in addition, their first choice included practical support and skills training related to living with ADHD. They saw this help and training as a way to become self-reliant. They did not want help that gave them a feeling of being outsiders or a failure. From Oliver’s perspective, managing ADHD symptoms with practical work is a better choice than experiencing failure with traditional education at school.

Many of those with ADHD, especially those who struggle with concentration, instead of sitting in school struggling behind a desk…they would benefit from learning in a practical way. What I am saying is that it is a much better solution to their problems to head out to learn things, practically early, instead of cramming them full of medicine.… If I did not have the diagnosis…well, I do not believe that an ordinary person would have been able to work so hard.…And, additionally, it’s damn important to always believe in yourself and never—never—never underestimate yourself…but at the same time, be a little humble. (Oliver)

Participants explained that they wanted to manage on their own and seek out their own resources, using experiences gained from living with ADHD from childhood to adulthood. Anna explained this preference as follows:

If it’s not okay, I’ll just take a day on my own and reflect over things, and what I can do about it.…Further, I can talk to my best friend and just get it out; then I am on track again, and it’s fine. I found tools and taught myself how to handle my ADHD early in life. (Anna)

Although all of the participants were interested in developing self-help strategies, they were in different phases of developing these strategies, and they described different experiences in terms of what strategies worked for them. If the problems were perceived as large, they could be more demanding, and, as a result, they fell short. This was something Julie experienced in connection with mastering her university studies. She wanted to master her studies but found that the organization of her studies in her everyday life became too difficult and made her feel lost:

I am a bit lost; I do not know what I should have done differently. (Julie)

Participants’ lived experiences partly helped them manage life on their own. They used various self-help strategies to manage adult life with ADHD; nevertheless, they saw there was still potential to develop these even more.

### A preference to discontinue medication use

Participants described that medical treatment was the first and, for many, only treatment offered by specialized mental health services. During the transition into adulthood, participants discontinued using medication or planned to quit because of side effects. The participants had different experiences, but all had experienced medical treatment as challenging. When they became adults, neither psychiatrists nor parents could control their decisions related to medication, and many of the participants chose to discontinue medication on their own, without involving anyone else. David still uses ADHD medication, but he has thought about terminating:

Actually, medicine or not…I still use it…but I try. This last year, I have tried to “detox” myself, and it has become my own project. (David)

Participants described that sessions with their family doctor (GP) were focused on effects of medication, and they were not asked about other aspects of living with ADHD symptoms:

After I turned 18…I went for medical controls once a year, but there was never focus on how I experienced living with the diagnosis, it was only about how the medication worked…I have stopped [taking medicine]; it did not work for me. (Anna)

In spite of the fact that most of the participants chose to discontinue medication use, some needed medication to function in school settings. However, Tom felt sorry that it was necessary to be medicated to manage school:

The fact that I had to be medicated to manage to get through school, I think that is a bit sad. (Tom)

Most of the participants experienced side effects, and this was often a reason why they discontinued medication. Phillip felt better when he stopped taking the medication, but, sometimes, he still wondered if medicine could have been helpful:

I felt better when I stopped, because I struggled with headaches, fatigue, and sleeping difficulties, and…things got a bit broken and gray. I was maybe somewhat depressed.…However, I could probably take medicine if there had been [any] without those side effects because, well, I’m a grown-up man. I should be able to realize when things go a little too fast and might calm down a bit. In those situations, it would have been better if I had not been hyper. (Phillip)

Many of the participants experienced feeling different about themselves and their personal beliefs using medication. Several, like Julie, explained that they somehow lost a part of their personality:

When I take medicine, I just…I feel like a completely different person…I do not feel I have that sparkle in my eyes. I notice that I become very monotonous in my voice, and my speech is in a lower tone, whereas without medicine, I am just more like, you know, sitting on the edge of my chair. (Julie)

According to the participants’ perspectives, medication was useful when they were younger, particularly in school. Even though they defined the benefit of medical treatment for their symptoms, the cost of side effects affected their decisions to terminate them.

## Discussion

The overall aim of this study was to gain increased knowledge of emerging adults’ experience of living with ADHD in the transition from adolescence to adulthood. Based on the findings of this study, we will discuss (1) the need for a psychosocial focus on emerging adults with ADHD in transition to adulthood and (2) the fact that the treatment offered to young adults does not satisfy their needs, in light of recovery theory and through alternative treatment interventions for young people with ADHD. 

### The need for psychosocial focus on emerging adults with ADHD in transition to adulthood

The participants in this study experienced a lack of psychosocial treatment options and expressed that the treatment they received was based on symptom reduction rather than finding a way to live a meaningful life with their symptoms. Thus, they communicated a need to learn more about ADHD and how to live and manage life with the diagnosis despite the challenges, which are central in mental health recovery [22]. In the recovery literature, a health-promoting focus is emphasized, and to be “in recovery” refers to the process of living one’s life using one’s strengths [22].

During the transition to young adulthood, most participants chose to discontinue medication, which is in line with previous studies [16, 17]. The participants used their own strategies based on previous experiences that they found helpful instead of ADHD medication, and, therefore, they found their “personal medicine” and developed a set of self-care skills to help manage and minimize mental health challenges [53]. Deegan (2005) described personal medicine as self-initiated, nonmedical self-care activities that serve to improve mood, thoughts, behaviors, and an overall sense of well-being.

The participants had varying experiences when it came to managing their ADHD symptoms in adulthood. Phillip expressed that he will never seek out help; he wanted to manage life by himself through his own strengths and claimed the rights to self-determination. This in line with the concept of “recovery in,” which refers to a person’s rights to self-determination and inclusion in community life despite continuing to suffer from mental illness [22]. Recovery does not require remission of symptoms or other deficits or a return to normal functioning. Rather, in recovery, mental illness is seen as only one aspect of an otherwise whole person [54]. However, perhaps Phillip suffered through his misery because of the lack of support; thus, he might have asked for help if he had access to the available support.

“There Is Time to Modernize the Concept of ADHD” is the title of a recent Editorial article by Franke (2023). This new concept takes the discourse on ADHD to the next level, as the author underlined, from a limited symptom and impairment-driven paradigm to a dynamic model, acknowledging weaknesses and strengths and self-management [55]. In such a paradigm, Phillip would have been given support to strengthen his self-management, whereas the professionals would have assumed the supporter role in his journey to find strategies useful to him. The participants in this study revealed a need for support systems and community care services they could identify with. When providing help from the specialized mental health services, the focus was on diagnosis, symptoms, and their limitations. Recovery from illness seemed to be the main focus, where the participants should recover from a biologically determined illness. This is in line with the understanding of clinical recovery, in the sense of a cure from all symptoms of mental illness [56, 57]. The participants described a more liberating imperative where they emphasized their own experience as important in their recovery process, not just patient deficits and professional expertise. This can be linked to recovery as a personal and social process, in which the individual’s own processes go hand in hand with resources from networks and local communities to support the individual to live their best possible life [24]. This does not mean that they will manage everything by themselves but that the support from others must be at a level that they can identify as helpful.

### The treatments offered to young adults do not satisfy their needs

The participants expressed a need for an alternative way of thinking about interventions over the course of their transition to adulthood, as they called for a different type of support. They talked about needing skills training and specific guidance on how to handle challenges in practical situations. Instead of using medication to manage school, they suggested training and work that were more practical: Social and vocational training, support, and guidance for people with mental health conditions can improve quality of life [25]. In spite of global and national guidelines [15], the participants were missing individual practical guidance in everyday life and wanted to know what to predict in every phase of life. This emphasizes a need for alternative interventions in the transition into adulthood [18]. Schrevel et al. (2016) suggested that strength-based coaching, which reinforces personal strengths and competences, could be a good option [58].

In the absence of knowledge and access to psychosocial interventions, the participants in this study used their own resources and tried to learn from previous experiences. However, the lack of quality-assured psychoeducation about ADHD from professionals led them to seek out information elsewhere. Consequently, the participants called for information on social media and digital platforms such as TikTok [59]. We must be aware that approximately half of TikTok videos about ADHD can be misleading [28], which underlines the need for professionals to be aware and possibly be more active on social media, such as TikTok, or interventions on the internet. Kenter et al. (2023) highlighted that interventions via the internet offer treatment outside of the traditional treatment centers, representing a low-intensity intervention population-wide.

The participants also spoke about the importance of peers in terms of exchanging information and accessing peers’ stories about living with ADHD, which gave them peer-to-peer support. Young people use the internet to seek out information and to communicate with peers, and internet support groups have been established for young people with mental health problems [60], which might be an alternative intervention for young people with ADHD with unmet needs. Participants suggested exchanging information with peers or joining groups, and several of them revealed that meeting others with ADHD had been beneficial. Fortunately, during the last couple of years, interventions such as group therapy for adolescents and young adults with ADHD have become more common [16]. In addition, low-threshold user groups have been established, and ADHD Europe pointed out that adults often feel there is no place to go for help with their daily life problems [4].

Participants in this study often hesitated to seek out help, worried that the health support system would offer treatment they did not want or think they need. When the kind of support they wanted or needed did not exist, one strategy might be to reject that they needed any support. In this study, the participants strived to determine their own strategies to handle symptoms and life in general. Nevertheless, they were aware that they had indeed needed some help from professionals. In line with Franke (2023), who proposed a paradigm shift, we argue that to reduce the gap between the help they are offered today and the help they themselves express a desire for, there is a need for a paradigm shift in the assessment and treatment of emerging adults.

## Conclusion

The participants in this study revealed that they did not seek out or had not been offered help during emerging adulthood. The treatment they had been offered was particularly linked to symptom reduction and medication use. However, they wanted to be guided on how to live with their diagnosis and their symptoms, preferring psychosocial interventions. As far as we know, up to the current day, research on emerging adulthood is lacking when it comes to psychosocial interventions.

Our study underlines that emerging adults living with ADHD may be particularly vulnerable during this transitional phase; however, they want to live meaningful lives, and we argue that psychosocial interventions can promote the well-being and recovery of emerging adults with ADHD. Nevertheless, they wanted alternative treatment and more practical help within easy reach, as they called for interventions such as practical skills training, digital interventions, group interventions, and peer-to-peer support.

### Implications

To meet the needs of young people with ADHD in the transition to adulthood, we recommend that helpers take notice of the recovery model when reaching out to young adults with ADHD. We have to meet the needs of young people with ADHD, “where they are”, and the support and treatment provided should be linked to what the emerging adults themselves highlight as important to a greater extent—in other words, the kind of support—so they may learn how to live meaningful lives, regardless of high or low symptom pressure. Such a focus is in line with health-promoting approaches to mental health, such as the recovery perspective. The recovery perspective takes a holistic view of a person’s life and how to live with their symptoms [54], promoting resilience and strength [61], and it could be helpful in understanding the needs of emerging adults with ADHD.

Emerging adults with ADHD wish for low-threshold interventions that focus on knowledge exchange and skills training, as well as for diverse follow-up and interventions, aimed directly at gaining knowledge about ADHD and how to live meaningful lives despite the diagnosis. We suggest digital interventions, which seem to fit their needs, and more professional information sharing in social media and digital health interventions aimed at emerging adults. This kind of help might not be perceived as stigmatizing, and therefore easier to seek out, during the transition into adulthood for young people living with ADHD.

The participants` experiences with the lack of information in the transition into young adulthood might be an important signal to clinicians working with adolescents and young adults with ADHD. Indeed, information on the transition process is important for clinicians supporting young adults living with ADHD [36]. When young people do not seek help from traditional specialized mental health services and instead choose to seek information about ADHD and how to manage the transition to adulthood from alternative sources, how can clinicians and helpers reach out to meet the needs of these young people in their everyday lives? We suggest that professionals develop qualified digital interventions that do not mislead and that are suited for young people with ADHD in the transition to adulthood.

Future mental health experts, professionals working with adolescents, and young people with ADHD should find alternative ways to raise awareness of the diagnosis. Interventions should be offered directly to these young people, and digital health interventions, including psychosocial interventions on how to manage life with ADHD, could be appropriate for young people with ADHD during the transition from adolescence into adulthood.

### Strengths and limitations

We recognize some strengths and limitations in this study. In qualitative research, researchers always have to think about reflexivity and the effect of context and investigators [47, 51].

The first author conducted the interviews on her own, and, therefore, there is a possibility that reflexivity may have been undermined. However, to strengthen the validity, Authors 1 and 3 read all of the transcriptions and met in several workshops to discuss the main themes. Author 2 read the meaning units in Step 3. In addition to strengthening the validity, Author 1 joined three workshops discussing the findings with the user organization (ADHD Norge) during the planning and writing processes.

There were more males than females in the sample, which might have affected the analyses, considering that we had more experiences from young men. Young et al. [62] declared that further research is needed to explore differences between males and females and to develop a better understanding of ADHD in girls and women.

### Electronic supplementary material

Below is the link to the electronic supplementary material.

Supplementary Material 1

## Data Availability

The qualitative datasets generated and analyzed during the current study are not publicly available due to limitations in the ethical approval, considering the responsibility to respect participants’ rights to privacy and to protect their identities. However, the corresponding author can provide transcript information on reasonable request.

## Declarations

### Authors’ information

The first author, Inger Lise Rasmussen, is an educational therapist in child and adolescent psychiatric services at Hospital of Namsos, Nord-Trøndelag Hospital Trust, and holds an academic degree, Cand.polit, from Norwegian University of Science and Technology: NTNU, Trondheim. The main research of interest are young people living with a diagnosis of ADHD and protective factors such as self-esteem, resilience, and recovery perspective.

The second author, Jorun Schei, MD, PhD, is a child and adolescent psychiatrist at St. Olavs University Hospital, Trondheim, and an associate professor at NTNU, Trondheim. The main research interests are neuropsychiatry, including ADHD, comorbidity, risk, and protective factors in a long-term perspective. Schei is the leader of the Health Survey in Department of Children and Youth, St. Olavs Hospital, a prospective longitudinal cohort study of psychiatric morbidity in an adolescent clinical population.

The third author, Kristin Berre Ørjasæter, is an associate professor in health and social sciences at Nord University, Faculty of Nursing and Health Science Namsos and leader of Mental Health Research Group at Nord University. She has a long-standing clinical experience as a therapist in specialized mental health services. The main research interests are mental health work, recovery-oriented practices, and citizenship.

## Abbreviations

ADHD: Attention deficit hyperactivity disorder
NAMI: National Alliance of Mental Illness
STC: Systematic text condensation
REK: Committee for Medical and Health Research Ethics (Regional Ethics Committee)
RFM: The Regional Academic Community for Autism, ADHD, and Tourette’s Syndrome
NTNU: Norwegian University of Science and Technology
DAC: The Research Department at Nord-Trondelag Hospital Trust
