# Supplementary material for: “A bit lost”—Living with attention deficit hyperactivity disorder in the transition between adolescence and adulthood: an exploratory qualitative study
Source: BMC Psychol. 2024 Jan 11;12:20. doi: 10.1186/s40359-024-01522-1 (PMC10785427; doi:10.1186/s40359-024-01522-1)
Supplement: Supplementary file 1 — Supplementary Material 1 [file 40359_2024_1522_MOESM1_ESM.docx]

**INTERVIEW GUIDE**

**Introduction**

Each interview starts with an introduction about the research project “ADHD in the transition between adolescent and adulthood.” This includes information about the project aim and the participant’s rights. All participants shall give written consent to participate before starting the interview. They will be told that they can withdraw from the project at any time.

**Overview of interview topics**

Experiences centered around living with ADHD

Experiences with education/school/work

Experiences with meaningful relationships

Experiences considering self-esteem

Experiences with coping/resilience

Experiences with help and treatment

The sub-questions will only be asked if the participants do not answer the main question.

**Questions**

**Tell me about yourself: who are you today?**

-What thoughts do you have about yourself and your self-esteem?

-How did you see yourself when you were younger, and how do you see yourself today?

**Tell me about what you are doing today?**

-Have you completed an education or started working?

-Tell me about your experiences with school/education or work?

-How have these experiences influenced your life?

**Tell me, do you have someone special who is close to you or important in your life?**

-Do you share your life with someone/do you have a partner?

-Have you started a family?

-Is there anyone who has meant a lot to you since you became an adult?

-Do you have any support persons who help you on a daily basis in some areas?

**How do you experience ADHD today?**

-Tell me about what it is like to live with ADHD as an emergent adult?

-In what way do you feel that the ADHD diagnosis affects/or has affected your life?

-What do you think has been important in getting you where you are today?

**Tell me a about your relation/contact with help services or treatment?**

-What type of help have you received?

-What type of help do you receive today?

-Have you received information and education about ADHD earlier or as a young adult?

-Have you called for help, or something else, that could have been important for you?

**What experiences do you have with the ADHD diagnosis today?**

-What problems are you struggling with today?

-How do you feel that these affect you or your life?

-Tell me about how you think others perceive you?

-Do you have concerns about your economy/wealth? In what way?

**How do you experience living with ADHD today?**

-Do you remember what you thought about having the diagnosis under the first interview, back in 2016?

-How do you experience living with ADHD as a grown up?

-Is there anything in your life that would have been different if you had not received the

ADHD diagnosis as a child?

**Do you have coping arenas in your life?**

-Tell me about your ambitions, further dreams, or hopes in life?

-Do you feel that you are mastering your life today, and in what way do you think that

your experiences have been mastered?

-Have you thought about what your most important strengths are?

**Do you currently use medication for ADHD?**

-What are your experiences with medication? How does it feel to use medication?

-If you do not use medication, can you tell me why?

**Finally:**

-Do you have other thoughts or experiences centered around living with ADHD in adulthood?

-Is there anything we have discussed that you would like to tell me more about?

**Conclusions and reflections in the conversation**

How do you feel about having this conversation?

Is there anything else you would like to add?

Thank you very much for your willingness to share your experiences .
